# Selective 5-HT7 receptor agonists LP 44 and LP 211 elicit an analgesic effect on formalin-induced orofacial pain in mice

**DOI:** 10.1590/1678-775720150563

**Published:** 2016

**Authors:** Kadriye DEMİRKAYA, Özlem Martı AKGÜN, Buğra ŞENEL, Zeynep ÖNCEL TORUN, Melik SEYREK, Enza LACİVİTA, Marcello LEOPOLDO, Ahmet DOĞRUL

**Affiliations:** 1 - Gulhane Medical Academy, Department of Restorative Dentistry and Endodontics, Ankara, Turkey.; 2 - Gulhane Medical Academy, Department of Pediatric Dentistry, Ankara, Turkey.; 3 - Gulhane Medical Academy, Department of Dentomaxillofacial Radiology, Ankara, Turkey.; 4 - Gulhane Medical Academy, Department of Pharmacology and Pain Research Centre, Ankara, Turkey.; 5 - Università degli studi di Bari Aldo Moro, Dipartimento di Farmacia – Scienze del Farmaco, Bari, Italy.

**Keywords:** Formalin, Receptor agonists, Orofacial pain

## Abstract

The most recently identified serotonin (5-HT) receptor is the 5-HT7 receptor. The antinociceptive effects of a 5-HT7 receptor agonist have been shown in neuropathic and inflammatory animal models of pain. A recent study demonstrated the functional expression of 5-HT7 receptors in the substantia gelatinosa (SG) of the trigeminal *subnucleus caudalis*, which receives and processes orofacial nociceptive inputs.

**Objective:**

To investigate the antinociceptive effects of pharmacological activation of 5-HT7 receptors on orofacial pain in mice.

**Material and Methods:**

Nociception was evaluated by using an orofacial formalin test in male Balb-C mice. Selective 5-HT7 receptor agonists, LP 44 and LP 211 (1, 5, and 10 mg/kg), were given intraperitoneally 30 min prior to a formalin injection. A bolus of 10 µl of 4% subcutaneous formalin was injected into the upper lip of mice and facial grooming behaviors were monitored. The behavioral responses consisted of two distinct periods, the early phase corresponding to acute pain (Phase I: 0–12 min) and the late phase (Phase II: 12–30 min).

**Results:**

LP 44 and LP 211 (1, 5, and 10 mg/kg) produced an analgesic effect with reductions in face rubbing time in both Phase I and Phase II of the formalin test.

**Conclusion:**

Our results suggest that 5-HT7 receptor agonists may be promising analgesic drugs in the treatment of orofacial pain.

## INTRODUCTION

Orofacial pain treatments remain an important consideration in dental care and patient management. Patients often evaluate a clinician’s ability by their success or failure in pain control. Dental pain management requires a multifactorial approach that includes a combination of good treatment procedures and the use of appropriate analgesics^12^.

The majority of drugs used to manage pain in Dentistry are nonsteroidal anti-inflammatory drugs (NSAIDs) and opioid analgesics^10^. Opioid analgesics are controlled substances and have many serious adverse side effects such as drug abuse, nausea, and vomiting^3^. Thus, most acute dental pain can be managed with the proper NSAID. However, subacute and chronic dental pain present difficulties due to the problems associated with the long term use of NSAIDs, which may be associated with significant side effects including gastrointestinal and renal diseases^17^. Thus, it is necessary to discover new drugs and new target molecules to relieve severe dental pain.

The 5-HT7 receptor was the lastly identified serotonin receptor subtype. Our previous studies and other studies show that 5-HT7 receptor play a key role both in endogenous pain control and exogenously administered opioid and non-opioid analgesics drug action^8^ ^,^ ^19^ ^,^ ^21^. There are very few studies that examine the analgesic effects of selective 5-HT7 receptor agonist. Brenchat, et al.^4^ ^,^ ^5^ (2010; 2012) reported that systemic administration of selective 5-HT7 agonists such as E-57431, AS-19, and E-55888 blocked nerve injury-induced mechanical and thermal hyperalgesia in rat. In inflammatory carrageenan model, Albayrak, et al.^1^ (2013) found that AS-19 elicits anti-inflammatory action with concomitant blockade of the increase in 5-HT7 receptor expression induced by intrapaw injection of carrageenan.

## MATERIAL AND METHODS

### Selection of the experimental group

Experiments were performed in the Drug Research and Development Laboratory of Pharmacology Department. Female Balb-C (25–32 g) mice were used. Mice were housed in a well-ventilated room at 22±2°C under a 12 h light: 12 h dark cycle and with free access to food and water. The experimental group consisted of 6–8 mice. Nociception was evaluated by using orofacial formalin test. Each of the subjects were allowed to separately provide adaptation within 30 min in the observation room in which a 30x30x30 cm Plexiglas box was placed with mirrors with a 45 degree angle to see unobstructed views of the orofacial region. The protocol was approved by the Animal Care and Use Committee.

### Drugs

Selective 5-HT7 receptor agonists, LP 44 and LP 211, were used for this study. While LP 44 was dissolved in distilled water, LP 211 was dissolved in 5% dimethyl sulfoxide (DMSO). LP 44 and LP 211 (1, 5, and 10 mg/kg) were given intraperitoneally (i.p.) in a volume of 5 ml/kg, 30 minutes prior to the formalin injection. Vehicle was given to the control groups.

### Orofacial formalin test and the assessment of antinociceptive effects

Orofacial nociception was evaluated using formalin as previously described^11^. A bolus of 4% formalin (10 µl) was subcutaneously injected into the upper lip of mice just lateral to the nose. The time of the paw strokes directed to the injection area, called face rubbing, was recorded over a 30-minute period, which was divided into 10 blocks of 3 min each, while the mice were in Plexiglas cages. In our study, cumulative data collected between 0 and 9 min post-formalin injection were grouped as phase 1 (0–9 min), whereas phase 2 was composed of data collected between 12 and 30 min post-formalin injection (the late phase).

### Statistical analysis

Data are expressed as means±standard error of the mean (SEM) for each group. GraphPad Prism 4 software (GraphPad Software, San Diego California, USA) was used for the statistical analyses. In the first stage, the time course effects of drug or vehicle treatment on face rubbing responses were evaluated by two-way repeated measure analysis of variance. Next, the averages of the period (0–9 min for Phase I and 12–30 min for Phase II) were calculated in each animals (bar graphs) and one way ANOVA were used to compare groups. A significant effect on the main factor(s) was taken as the criterion for progressing to *post-hoc* testing. The Bonferroni *post-hoc* test was used to compare the groups. Statistical significance was accepted as *p*<0.05.

## RESULTS

**Figure 1:**
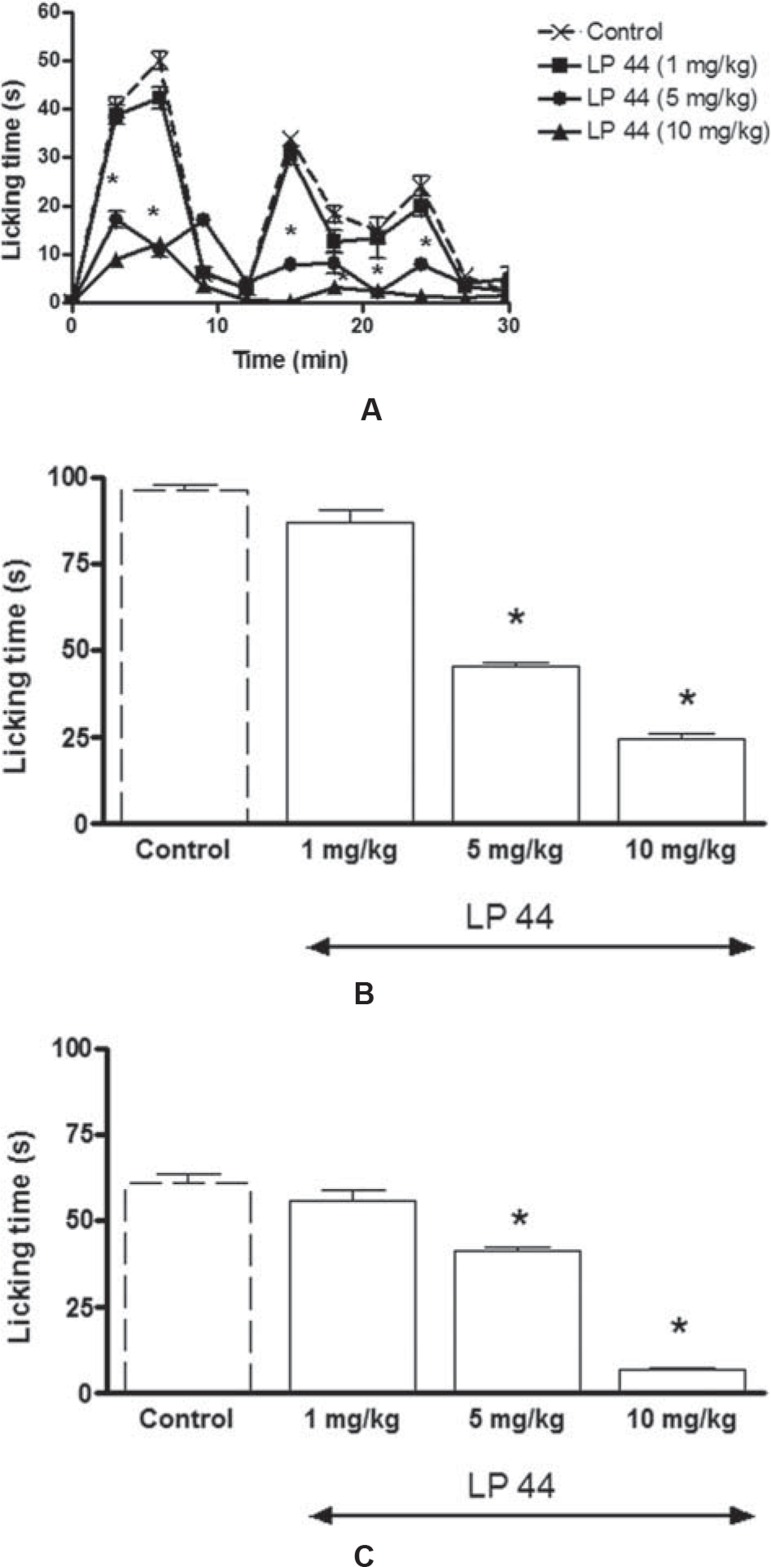
Time course of face rubbing activity induced by formalin injection into upper lip after systemic administration of LP 44, a selective 5-HT7 receptor agonist, or vehicle. Mice were pretreated with systemic administration of LP 44 (1, 5, and 10 mg/kg), 30 min prior to injection of 4% formalin (10 µl) into upper lip (A). Bar graphs represent the mean cumulative face rubbing time in s at Phase I (0–9 min) (B) and Phase II (12–30 min) (C). * P<0.05, differences from control groups (Bonferroni *post hoc* test)

Consistent with previous studies, following the formalin injection, a typical biphasic time course with an early and short-lasting (9 min) first period of activity (Phase I) was observed. This was followed by a 3-minute quiescent period. Then, a second, prolonged (12–30 min) tonic phase (Phase II) was observed (Figure 1A). The mean of the total face rubbing times were 96.29±1.6 seconds and 61.33±2.41 seconds during Phase I (0–9 min) and Phase II (12–30 min), respectively (Figures 1B and 1C).

The two-way repeated measures of ANOVA analysis indicated that systemic administration of LP 44 had significant effect on face rubbing responses induced by formalin injection (F_3,392_=601.1, *P*<0.0001) (Figure 1A). While 1 mg/kg was ineffective, LP 44 doses of 5 and 10 mg/kg profoundly reduced face rubbing times at 3 and 6 min in Phase I (Figure 1A). Similar to Phase I, LP 44 at doses of 5 and 10 mg/kg significantly inhibited formalin-induced face rubbing responses at 15, 18, 21, and 24 min in Phase II (Figure 1A). The mean of the total face rubbing times in Phase I, after LP 44 injection at the doses of 5 and 10 mg/kg, were significantly reduced to 45.5±1.08 and 24.8±1.3 s (both p<0.001), respectively, in Phase I, which were significantly different from control groups (Figure 1B). LP 44 at the doses of 5 and 10 mg/kg also significantly reduced the total face rubbing times to 41±1.08 and 7.3±0.7 s (both p<0.01), respectively, in Phase II, when compared with the control group (Figure 1C).

**Figure 2:**
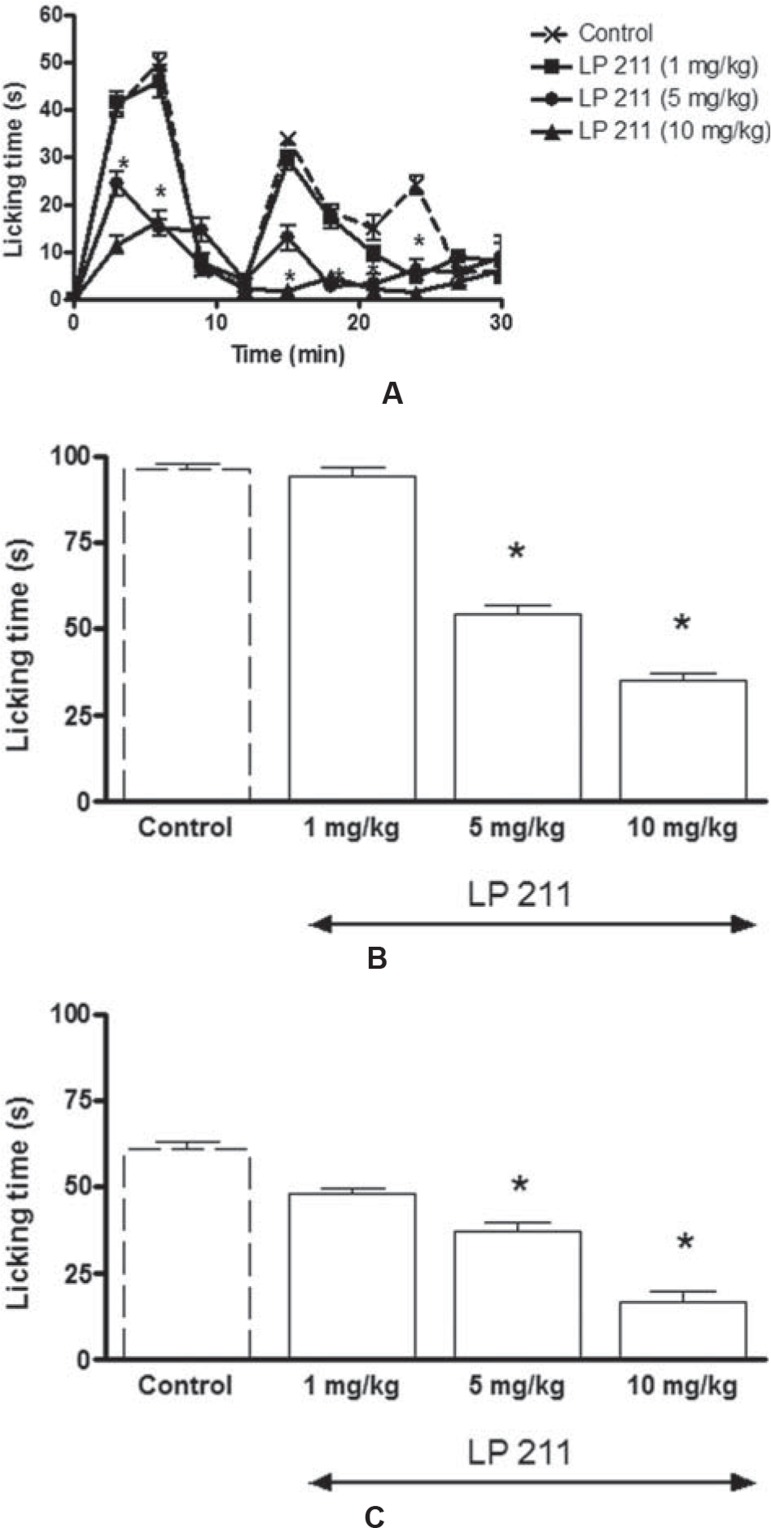
Time course of face rubbing activity induced by formalin injection into upper lip after systemic administration of LP 211, a selective 5-HT7 receptor agonist, or vehicle treatment. Mice were pretreated with systemic administration of LP 211 (1, 5, and 10 mg/kg), 30 min prior to injection of 4% formalin (10 µl) into upper lip (A). Bar graphs represent the mean cumulative face rubbing time in s at Phase I (0–9 min) (B) and Phase II (12–30 min) (C). * P<0.05, differences from vehicle groups (Bonferroni *post hoc* test)

Similar to LP 44, systemic administration of LP 211 had significant effect on formalin-induced face rubbing responses in Phase I and II of the formalin test (F_3,392_=213.77, *P*<0.0001) (Figure 2A). While LP 211 at a dose of 1 mg/kg did not alter formalin-induced nociceptive face rubbing responses, LP 211 doses of 5 and 10 mg/kg significantly reduced face rubbing times at 3 and 6 min in Phase I (Figure 2A). The antinociceptive effects of LP 211 at the doses of 5 and 10 mg/kg were evident at 15, 18, 21, and 24 min in Phase II when compared with the vehicle group (Figure 2A). The mean of face rubbing times at the 5 and 10 mg/kg of LP 211 pretreated groups in Phase I were reduced to 54.6±2.8 and 35.3±2.28 seconds (both p<0.001, respectively, which were significantly different from the vehicle-treated group (Figure 2B). Similarly, LP 211 at doses of 5 and 10 mg/kg also significantly decreased face rubbing times during Phase II to 37.2±2.7 and 17.88±2.94 s (both p<0.001), respectively, when compared with 5% DMSO-treated vehicle group (Figure 2C).

## DISCUSSION

In this study, we have demonstrated that selective 5-HT7 receptor agonists elicit analgesic effects on orofacial pain. Our data confirm previous studies implying a role of 5-HT7 receptors in pain pathways.

The pathologic states of the teeth and the related structure induced acute orofacial pain is one of the most common pain, and it is well known that the orofacial region is heavily innervated by the trigeminal nerve. The orofacial formalin test is a recognized model of animal model of nociception related to trigeminal pain^13^. Formalin, as an electrophile irritant, induces tissue injury following its intracutaneous injection accompanied with prototypical a biphasic pattern of pain response^6^. The early phase is generally attributed to a direct effect of formalin on the nociceptors, while the late phase appears to be dependent on an inflammatory reaction in the peripheral tissues and functional changes in spinal and brain stem spinal cord^6^. In the orofacial formalin test, face rubbing with the ipsilateral forepaw due to formalin injection into the upper lip has been mentioned as a specific nociceptive response^18^.

The serotonin in peripheral, spinal and supraspinal sites play in important role in the regulation of pain. Following tissue injury or inflammation, a variety of mediator or modulators together with 5-HT are released that activate or sensitize nociceptors^8^. It has been hypothesized that 5-HT, functioning in combination with other inflammatory or algesic mediators significantly modulate pain transmission in peripheral and central nervous system. 5-HT modulates the pain via its receptors. Several different subtypes of 5-HT receptors have been suggested to be involved in pain transmission and pain modulation^19^. However, the multiplicity of 5-HT receptors and the complex circuitry of pain transmission in peripheral and central nervous system complicates the analysis of subtypes of 5-HT receptors involved in the analgesia.

The 5-HT7 receptor was the lastly identified serotonin receptor subtype. Our previous studies and other studies show that 5-HT7 receptor play a key role both in endogenous pain control and exogenously administered opioid and non-opioid analgesics drug action^8^ ^,^ ^19^ ^,^ ^21^. There are very few studies that examine the analgesic effects of selective 5-HT7 receptor agonist. Brenchat, et al.^4^ ^,^ ^5^ (2010; 2012) reported that systemic administration of selective 5-HT7 agonists such as E-57431, AS-19, and E-55888 blocked nerve injury-induced mechanical and thermal hyperalgesia in rat. In inflammatory carrageenan model, Albayrak, et al.^1^ (2013) found that AS-19 elicits anti-inflammatory action with concomitant blockade of the increase in 5-HT7 receptor expression induced by intrapaw injection of carrageenan.

The important finding of this study is that the intraperitoneal administration of the selective 5-HT7 receptor agonists LP 44 or LP 211 produced an antinociceptive activity in both phases of the orofacial formalin test. This study expands the previous studies indicating the analgesic effects of 5-HT7 receptor agonist in other animal models of pain as to the efficacy of this group of drugs on orofacial pain. In line with previous reports describing anti-inflammatory and analgesic action of 5-HT7 receptor agonists under sensitization painful condition^5^, as expected, we found that LP 44 and LP 211 reduced the nociceptive behavior in both phases of the orofacial formalin test. As CNS-penetrant drugs, after systemic administration, LP 44 and LP 211 can access peripheral, spinal, and supraspinal sites^15^. Thus, in the present study, the site of action underlying analgesic effects of 5-HT7 agonist is unclear. Several studies demonstrated the involvement of descending serotonergic systems in modulating the orofacial nociception^7^. Interestingly, we reported the special important role of 5-HT7 in the descending pain inhibitory pathways^7^ ^,^ ^21^. Neuroanatomical studies demonstrate the existence of 5-HT7 receptors in the *substantia gelatinosa* of the trigeminal *subnucleus caudalis*, which play a critical role in mediating orofacial nociception. Thus, it is possible to speculate that the antinociceptive action of LP 44 and LP 211 may be attributed to the activation of 5-HT7 receptors in the *substantia gelatinosa* neurons of the trigeminal nerve. Nevertheless, we cannot exclude the possibility that 5HT2A receptors contribute to antinociception in the other brain areas and/or in the periphery. Further studies are needed to clarify the exact mechanism of analgesic action of 5-HT7 receptor agonist on orofacial pain.

## CONCLUSION

In conclusion, our results suggest that targeting 5-HT7 receptor might provide a new therapeutic tool for the treatment of orofacial painful conditions. However, the efficacy of 5-HT7 receptor agonist should be tested in human orofacial pain models.
